# Automatic Classification of Adult Males With and Without Autism Spectrum Disorder by Non-contact Measurement of Autonomic Nervous System Activation

**DOI:** 10.3389/fpsyt.2021.625978

**Published:** 2021-05-17

**Authors:** Hirokazu Doi, Norimichi Tsumura, Chieko Kanai, Kenta Masui, Ryota Mitsuhashi, Takumi Nagasawa

**Affiliations:** ^1^Graduate School of Engineering, School of Science and Engineering, Kokushikan University, Setagaya, Japan; ^2^Graduate School of Biomedical Sciences, Nagasaki University, Nagasaki, Japan; ^3^Graduate School of Engineering, Chiba University, Chiba, Japan; ^4^Medical Institute of Developmental Disabilities Research, Showa University, Tokyo, Japan; ^5^Faculty of Humanities, Wayo Women's University, Chiba, Japan

**Keywords:** ASD, autonomic nervous system, emotion, non-contact measurement, digital phenotyping, pulse wave, color

## Abstract

People with autism spectrum disorder (ASD) exhibit atypicality in various domains of behavior. Previous psychophysiological studies have revealed an atypical pattern of autonomic nervous system (ANS) activation induced by psychosocial stimulation. Thus, it might be feasible to develop a novel assessment tool to evaluate the risk of ASD by measuring ANS activation in response to emotional stimulation. The present study investigated whether people with ASD could be automatically classified from neurotypical adults based solely on physiological data obtained by the recently introduced non-contact measurement of pulse wave. We video-recorded faces of adult males with and without ASD while watching emotion-inducing video clips. Features reflective of ANS activation were extracted from the temporal fluctuation of facial skin coloration and entered into a machine-learning algorithm. Though the performance was modest, the gradient boosting classifier succeeded in classifying people with and without ASD, which indicates that facial skin color fluctuation contains information useful for detecting people with ASD. Taking into consideration the fact that the current study recruited only high-functioning adults who have relatively mild symptoms and probably developed some compensatory strategies, ASD screening by non-contact measurement of pulse wave could be a promising assessment tool to evaluate ASD risk.

## Introduction

Autism spectrum disorder (ASD) is a group of closely related developmental disabilities characterized by a collection of symptoms including persistent deficits in social communication and reciprocal interaction, repetitive behavior, and restricted interest (1). Although genetics is considered to play major role in the development of ASD, despite years of effort by researchers, the exact cause of ASD still remains elusive (2). As such, there exists no definitive cure for ASD.

Public awareness of ASD has risen significantly in recent years (3), but several surveys suggest the possibility that a significant portion of the ASD population remains undiagnosed (4). Importantly, some of them remain undiagnosed well into adulthood and experience great distress due to failure in adaptation to occupational and social environments (5–7). The difficulty in detecting ASD risk in adults is attributable to several causes. First, adults with milder cases of ASD can sometimes compensate for their atypicality to some degree by adjusting their behavior with deliberate effort. Second, misdiagnosis is prone to be made because of the comorbidity of other psychiatric conditions.

One promising way to reduce the number of undiagnosed adults with ASD is to develop a supplementary assessment tool for ASD. The currently accepted protocol for ASD diagnosis requires hours of interviews and assessment by clinicians and is quite labor-intensive. Such strict protocols are relevant from the perspective of suppressing overdiagnosis of ASD. At the same time, it is also necessary to establish a convenient and handy assessment tool for primary screening to broadly catch those who “might” have ASD.

Recent studies on digital phenotyping (8) have pointed to the potential of semiautomated quantification of behavioral phenotypes in the primary screening of ASD. In these studies, behavioral features of people with ASD were captured by information technology, such as video analysis of facial movement by computer vision (9–11) and bodily movement analysis using cost-effective sensors [Kinect sensors and touch sensors implemented in tablet computers; (12, 13)]. Some studies have entered the extracted features into machine learning and succeeded in classifying ASD and neurotypical counterparts (11–13).

Classical theories of ASD have claimed that atypicality in emotional function causes ASD-like behaviors (14, 15). Although not in its original form, some of these theories have gained support from studies showing poor performance in emotion recognition (16, 17) and atypical patterns of neural activation and morphology in emotion-associated regions such as the amygdala (14, 18). In addition to atypical development of emotion-related neural regions, a number of studies indicate that people with ASD, especially pediatric patients, show poor ability of emotional regulation (19) as can be seen in occurrences of sudden burst of emotion, so-called “meltdown.” Taken together, people with ASD have atypicality in both the induction of emotion and emotional regulation, which leads to an atypical pattern of emotional responses.

Taking such atypical emotional responses in ASD people into consideration, measurement of emotional response might be useful in screening adults with ASD. Emotional response can be objectively quantified by measuring the activation of the autonomic nervous system (ANS), which comprises the sympathetic and parasympathetic nervous system. Psychophysiological studies have linked the relative increase of sympathetic over the parasympathetic nervous system to a high arousal state (20). In addition, several studies have found atypical patterns of ANS activation in people with ASD, especially in response to social situations. For example, studies have revealed reduced sympathetic activation in adults with ASD in response to social interaction (21) and psychosocial stress (22). Similar atypicality has also been observed in pediatric samples (23), although the exact pattern of atypicality differs among studies (24, 25). Previous studies on ANS activation in people with ASD also revealed increased heart rate and reduced respiratory sinus arrhythmia during rest (26, 27) and sleep (28) in individuals with ASD.

Currently, measurement using a polygraph system is deemed the gold standard for ANS activity measurement. However, polygraph systems are relatively expensive and thus difficult to introduce in many facilities. In addition, attachment of sensors onto the skin surface makes people with ASD, especially those with hypersensitivity to tactile stimulation (29), uncomfortable. A recently introduced method of non-contact measurement of pulse waves (30, 31), an index of ANS activation, could be an alternative technique to a polygraph. In this method, the timing of the pulse wave is detected by analyzing a slight change in facial skin coloration. Immediately after cardiac pulsation, arterial blood rich in fresh-red oxygenated hemoglobin circulates through the blood vessels. Hemoglobin pigmentation on the skin surface is slightly intensified synchronously with this event. The temporal interval between neighboring peaks of pigmentation intensity reveals the balance between sympathetic and parasympathetic nervous system activations (32, 33) and, thus, certain aspects of emotional response.

The present study examined whether this non-contact measurement of ANS activation is assistive in distinguishing between neurotypical adults and adults with ASD. We extracted features reflective of ANS activation from video-recorded images of the participant's face while experiencing film-induced emotions (34, 35). These features were fed to machine-learning algorithms to ascertain whether pulse waves extracted from video-recorded images contain enough information to distinguish ASD adults from neurotypical ones. We focused on the phasic change of ANS activation during emotion-inducing film viewing from baseline.

## Method

### Participants

A total of 21 typically developed (TD) adult males (*M* = 31.7 years old; SD = 6.03) and 17 adult males with ASD (*M* = 31.5 years old; SD = 5.2) participated in the present study after giving written informed consent. We excluded female participants so as not to increase variance in responses in the ASD group because previous studies have found phenotypic differences between males and females with ASD (36, 37). The TD participants were recruited by word of mouth and recruitment agency. They had no ties or relations with the ASD participants. The average age did not differ significantly between the ASD and TD groups, *t*_(36)_ = 0.07, *p* = 0.94. All participants with ASD were referred to Showa University Hospital by physicians from other clinics. The inclusion criteria of the present study were as follows: males, Wechsler Adult Intelligence Scale-III, full intelligence ≥70 (high functioning evaluated by WAIS), and a formal diagnosis of ASD based on DSM-5 (1). The exclusion criteria were comorbid personality disorders. Three of them had a comorbidity of mood disorders. Out of 17 ASD participants, nine were on medication (antidepressant, *n* = 5; anxiolytic, *n* = 4; soporific, *n* = 3; antipsychotic, *n* = 4; anticonvulsant, *n* = 3). None was taking behavioral therapy. A psychiatrist made a diagnosis according to the diagnostic criteria of the DSM-5 for ASD based on a consensus among a team comprised of experienced psychiatrists and a clinical psychologist who carried out clinical interviews. All participants satisfied the Autism Diagnostic Observation Schedule diagnostic criteria at the time of the interview (*M* = 11.4, SD = 2.8). The diagnosis was reconfirmed after at least a 2-month follow-up period. The protocol of this research was approved by the ethical committees of the Graduate School of Biomedical Sciences in Nagasaki University and the Graduate School of Engineering in Chiba University, according to the Declaration of Helsinki.

### Apparatus and Stimuli

The stimuli included six video clips with lengths of about 1.5–4.5 min intended to induce happy, sad, fearful, and neutral emotional states. These video clips were taken from a TV show or films to induce these emotional states, the details of which are described in Table 1. Four of the video clips depicted social interactions among several people. Two more video clips, that is, popular line drawing animation and a video clip of factory machines without human figures, were included in the stimulus set because previous studies have indicated that some people with ASD are prone to show interest in these contents (38, 39). Due to the scarcity of studies on emotion induction by films in Japanese samples, a new set of emotion-inducing films was created for the present study. We first screened Asian films (most of them, Japanese ones) that seemed suitable for emotion induction. Scenes including graphic content and violence were avoided so as not to elicit shock from the participants. Then, a small pilot study was carried out in which our lab members (one female and five males) evaluated 13 Asian films^1^ intended to induce sad (two films), fear (three films), happy (two films), and neutral (two films) emotions and two machine factory scenes (two films). Based on the selectivity and intensity of emotional state evaluation, we chose the six films summarized in Table 1.

**Table 1:** Detailed descriptions of scenes in stimulus video clips.

**Clip**	**Film title**	**Scene description**
Happy	Gottu Ee Kanji (Japanese TV comedy show)	A female police officer expresses her intention to resign from the police force, but her boss refuses to accept the resignation. Their colleagues dressed in flamboyant costumes make a fuss of the situation.
Fear	The EYE (1995)	A female takes an elevator. She sees an old man in the elevator, but his image is not captured in the security camera. As the elevator ascends, the man, floating on the floor, moves toward the female.
Sad	A Cicada of the Eighth Day (2011)	A female fugitive is trying to escape an island by ferry with a hostage child, whom she brought up as her daughter. After realizing that they are surrounded by police officers, she tells the child to leave. Police officers arrest her and the child cries out for her.
Neutral	A Scene at the Sea (1991)	A couple boards a bus, but the driver cautions the man not to bring a surfboard into the bus. The bus leaves and the female keeps watching his boyfriend, who is standing in the bus stop with his surfboard, through the window of the bus.
Line drawing animation	Pendulum (2014)	A pendulum clock is drawn as a motif. Together with the pendulum swing, the animation shows snippets of the life of a couple. When the couple reaches the end of their life, the husband tries to reverse the swing of the pendulum, but the wife gently stops him. All the objects and people are drawn in a thick black line on a white background in this animation.
Machine factory	None	This video describes the movements of various factory machines and mechanical tools. No images of humans are included.

**Figure 1:**
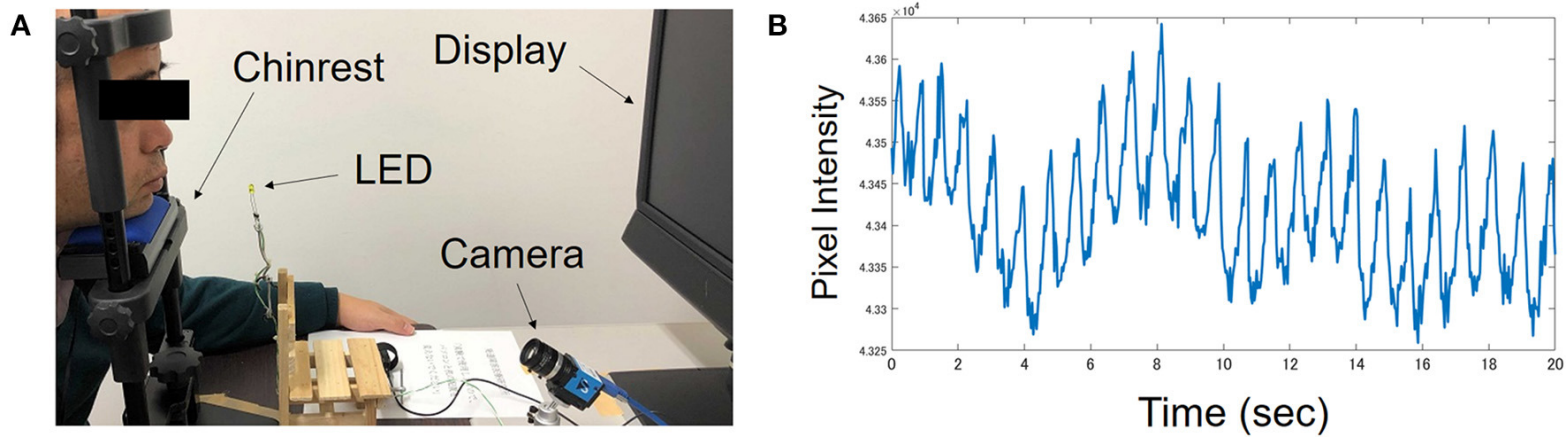
**(A)** A picture of experimental setting taken during preparation. The man in the picture is one of the authors, not a participant. During the actual experiment, participant held a keypad with their head stabilized on a chin rest. **(B)** Typical pattern of pulsatile fluctuation in hemoglobin intensity recorded from one ASD participant.

The participants viewed a 19-inch monitor with their head stabilized on a chin rest. Their faces were lit up using two fluorescent lights. The viewing distance was 75 cm. An LED light located near the participant's chin lit up at the start and the end of each video clip. The participants' faces and the LED lights were recorded by an RGB camera (situated between the monitor and the chin rest) for industrial use (ARGO Corporation, Suita, Osaka, Japan). A picture of the experimental setup is shown in Figure 1A.

### Procedure

#### Face Movie Recording

At the start of each trial, a black background was shown for 40 s. Then, a video clip was presented. The video clip included 10 s presentation of a brief explanation about the context of the scene. The participants were instructed to view the video clip without overt movement quietly. Immediately after the end of the video clip, seven track bars with 11 tics were presented on the screen. The participants' task was to answer their emotional states, that is, the levels of happiness, anger, sadness, fear, disgust, unpleasantness, and arousal, using the track bar. After the completion of the subjective evaluation of emotional state, the next trial started. Six video clips were separated into three blocks with interblock rest intervals. The order of the video clips was determined randomly.

#### Self-Administered Questionnaires

After the completion of the facial movie recording, the participants answered a battery of self-administered paper-and-pencil questionnaires in front of the experimenter. The questionnaires included Japanese translations of the IRI [Interpersonal Reactivity Index; (40)] and TAS-20 [The 20-item Toronto Alexithymia Scale; (41)]. The IRI measures empathic tendencies, and it comprises four subscales: perspective-taking, fantasy, empathic concern, and personal distress (42), while TAS measures the level of difficulty in identifying feelings, difficulty in describing feelings, and externally oriented thinking (41, 43).

### Image Processing

Recorded movies of the participants' faces were analyzed offline according to the algorithm described by Fukunishi et al. (31, 44). The skin color of the small facial region near the cheekbone was analyzed. First, skin coloration in each frame was separated into hemoglobin pigmentation, melanin pigmentation, and shade information by independent component analysis (45). Because shade information is separated from hemoglobin intensity, the intensity of hemoglobin pigmentation can be assessed independently of lighting condition. Then, the temporal fluctuation of hemoglobin intensity across video frames was analyzed.

The temporal course of hemoglobin pigmentation intensity shows pulsatile fluctuation as shown in Figure 1B. From the temporal course of hemoglobin pigmentation fluctuation, 20 indices of ANS activation were computed for each video clip for each participant. The details of the extracted indices are summarized in Table 2. These indices were measured during 30 s baseline just before video clip presentation and during video clip viewing. We used Kubios-HRV software (46) for computing some of the indices. The phasic change in these indices from baseline was calculated by subtracting index values during baseline from those during video clip viewing. Thus, six change-from-baseline values were obtained for each of the 20 indices, yielding a total of 120 features for machine learning.

**Table 2:** Summary of extracted ANS activation indices.

**Index no**.	**Description**
1	LF/HF by the FFT spectrum: ratio between LF (0.04–0.15 Hz) and HF (0.15–0.4 Hz) band power evaluated by Fourier transformation (FFT)
2	LF/HF by the AR spectrum: ratio between LF and HF band power evaluated by autocorrelation (AR)
3	Normalized LF power by FFT: LF band power normalized by total power minus very low frequency (VLF) band power evaluated by FFT
4	Normalized HF power by FFT: HF band power normalized by total power minus very low frequency (VLF) band power evaluated by FFT
5	Normalized LF power by the AR spectrum: LF band power normalized by total power minus very low frequency (VLF) band power evaluated by AR
6	Normalized HF power by the AR spectrum: HF band power normalized by total power minus very low frequency (VLF) band power evaluated by AR
7	Relative LF power against total power by the FFT spectrum: ratio between LF band power and total power evaluated by FFT
8	Relative HF power against total power by the FFT spectrum: ratio between HF band power and total power evaluated by FFT
9	Relative LF power against total power by the AR spectrum: ratio between LF band power and total power evaluated by AR
10	Relative HF power against total power by the AR spectrum: ratio between HF band power and total power evaluated by AR
11	Mean of RRI: mean of RR interval
12	Mean of HR: mean heart rate
13	RMSSD: root mean square of differences of successive RR intervals
14	pNN50: number of successive RR intervals that differ more than 50 ms divided by the total number of RR intervals
15	HRV triangular index: area covered by RR interval histogram divided by the height of the histogram
16	TINN: bottom width of RR interval histogram
17	SD1 of Poincaré plot: standard deviation of data points along the line of identity of an ellipsoid approximating the Poincaré plot (a scatter plot with *n-*th RR interval as horizontal axis and *n* + 1-th RR interval as vertical axis)
18	SD2 of Poincaré plot: standard deviation of data points in the direction perpendicular to the line of identity of an ellipsoid approximating the Poincaré plot
19	Ratio between SD1 and SD2: ratio between SD1 and SD2 in the Poincaré plot
20	Mean of hemoglobin intensity: mean pixel value of hemoglobin image

*The definitions summarized above are partly taken from the site of Kubios software (https://www.kubios.com/downloads/Kubios_HRV_Users_Guide.pdf)*.

*FFT, Fourier transformation; LF, low frequency; HF, high frequency; AR, autoregression; RRI, RR interval; HR, heart rate; RMSSD, root mean square of successive differences between heartbeats; pNN50, percentage of neighboring NN intervals (interval between normal heartbeats) that differ from each other by more than 50 ms; HRV, heart rate variability; TINN, triangular interpolation of RR intervals; SD, standard deviation*.

### Group Classification by Machine Learning

To ascertain whether participants with and without ASD can be automatically classified by 120 features reflective of ANS activation obtained by non-contact measurement, data were analyzed using a supervised machine-learning algorithm. First, 120 features were standardized and then compressed to principal components (PCs) by principal component analysis (PCA). Then, the scores of PCs were fed into a gradient boosting (GB) classifier. GB is one of the ensemble learning algorithms, which is known to show good prediction performance and generalizability, i.e., the ability of the classifier to predict the class of unknown data, over the other types of machine learning algorithms in many classification problems. In ensemble learning, a set of weak predictors with limited prediction ability is created, instead of a single strong predictor with high prediction ability. Then, the final prediction is made by aggregating the predictions of these weak predictors. Intuitively, in GB, weak predictors are added sequentially to the decision trees so that the addition of a new predictor gradually minimizes the discrepancy between observed data and prediction. There are two popular ensemble learning algorithms, GB and random forest. Though random forest excels at generalizability in some cases, GB can show superior performance when its hyperparameters are tuned appropriately.

The GB classifier was cross-validated by a leave-one-out procedure. Specifically, data from one participant were used as the unknown test data, and the data of the remaining 37 participants were used to train the classifier. After training, the classifier predicted which group, i.e., TD or ASD, the unknown test data belonged to. The performance indicators, i.e., accuracy, sensitivity, specificity, precision, and F1 score, were calculated according to the formula below after repeating this procedure 38 times by using the data of every participant as test data.

$$\begin{array}{l} \text{Accuracy}=\;\frac{\text{TP}+\text{TN}}{\text{TP}+\text{FP}+\text{TN}+\text{FN}}\\ \text{ Sensitivity}=\;\frac{\text{TP}}{\text{TP}+\text{FN}}\\ \text{ Specificity}=\;\frac{\text{TN}}{\text{TN}+\text{FP}}\\ \text{ Precision}=\;\frac{\text{TP}}{\text{TP}+\text{FP}}\\ \text{F}1\text{ Score}=\;\frac{2\times \text{Sensitivity}\times \text{Precision}}{\text{Sensitivity}+\text{Precision}} \end{array}$$

where TP (true positive), FP (false positive), TN (true negative), and FN (false negative) represent the frequencies with which the ASD participant was correctly identified as ASD, the TD participant was misclassified as ASD, the TD participant was correctly classified as TD, and the ASD participant was misclassified as TD, respectively.

To obtain a robust estimate of performance, we repeated this process 20 times by changing the random number seed.

### Basic Emotion Classification by Machine Learning

Among the six video clips, happy, fear, sad, and neutral clips are intended to induce a particular emotional state. In order to examine whether these four clips induced differential physiological states in ASD and neurotypical adults, a GB classifier was trained to discriminate these four clips based on the 20 ANS indices. The features were first compressed by PCA, and then the GB classifier was trained for the TD and ASD groups separately.

In the ASD group, there were emotional category (4) × participant (17) = 68 data. In the leave-one-out procedure, one of the 68 data was treated as unknown test data and the GB classifier was trained on the basis of the remaining 67 data. Accuracy rate and confusion matrix were computed after repeating this procedure 68 times using every one of the 68 data as the test data. Accuracy and confusion matrix were computed in the TD group according to essentially the same procedure using emotional category (4) × participant (21) = 84 data. Computation of accuracy and confusion matrix was repeated 20 times by changing the random seed.

In the next step of analysis, we tested whether emotional states are less clearly differentiated in ASD than TD participants (47). If this hypothesis is correct, the pairwise similarities among physiological responses induced by the four emotion-inducing clips should be higher in the ASD group than in the TD group. To examine this possibility, the cosine similarity of physiological states induced by the four video clips was calculated based on the confusion matrices. The cosine similarity between emotion *j* and emotion *k*, *CS*_*jk*_, is computed according to the following formula where *N*_*ij*_ represents the frequency with which emotion *j* was classified as emotion *i* (happy, fear, sad, neutral).

$$\begin{array}{l} CS_{jk}=\;\frac{\sum_{i=1}^{4}N_{ij}\times N_{ik}}{\sqrt{\sum_{i=1}^{4}N_{ij}^{2}}\times \sqrt{\sum_{i=1}^{4}N_{ik}^{2}}} \end{array}$$

### Statistical Analysis

Subjective ratings of emotional states were entered into an analysis of variance (ANOVA) with the between-participant factor of group (2; TD, ASD) and the within-participant factors of clip (6; happy, fear, sad, neutral, line drawing, machine) and emotional state (7; happiness, anger, fear, sadness, disgust, surprise, unpleasantness, arousal). When an interaction reached significance, simple main effect analysis was conducted to clarify its source.

In leave-one-out, the GB classifier gives the probability estimate that the test data belong to the ASD class. In the present study, leave-one-out cross-validation was repeated 20 times, yielding 20 probability values for each participant. We tested whether the average probabilities assigned to ASD participants were higher than those assigned to TD participants as expected by the Mann–Whitney *U*-test. The chi-squared test was used to examine if the proportions of participants classified into the ASD group differed between the ASD and TD groups. It was tested whether the mean accuracy of classification was significantly above chance (50%) by two-tailed *t*-test.

Group difference in subscores of IRI and TAS-20 was tested by two-tailed *t*-tests. To see if these traits are associated with classification results by the GB classifier, Spearman's rank-order correlation was tested between averaged probabilities and the scores of IRI and TAS-20.

## Results

### Group Difference in Subjective Emotional States

**Figure 2:**
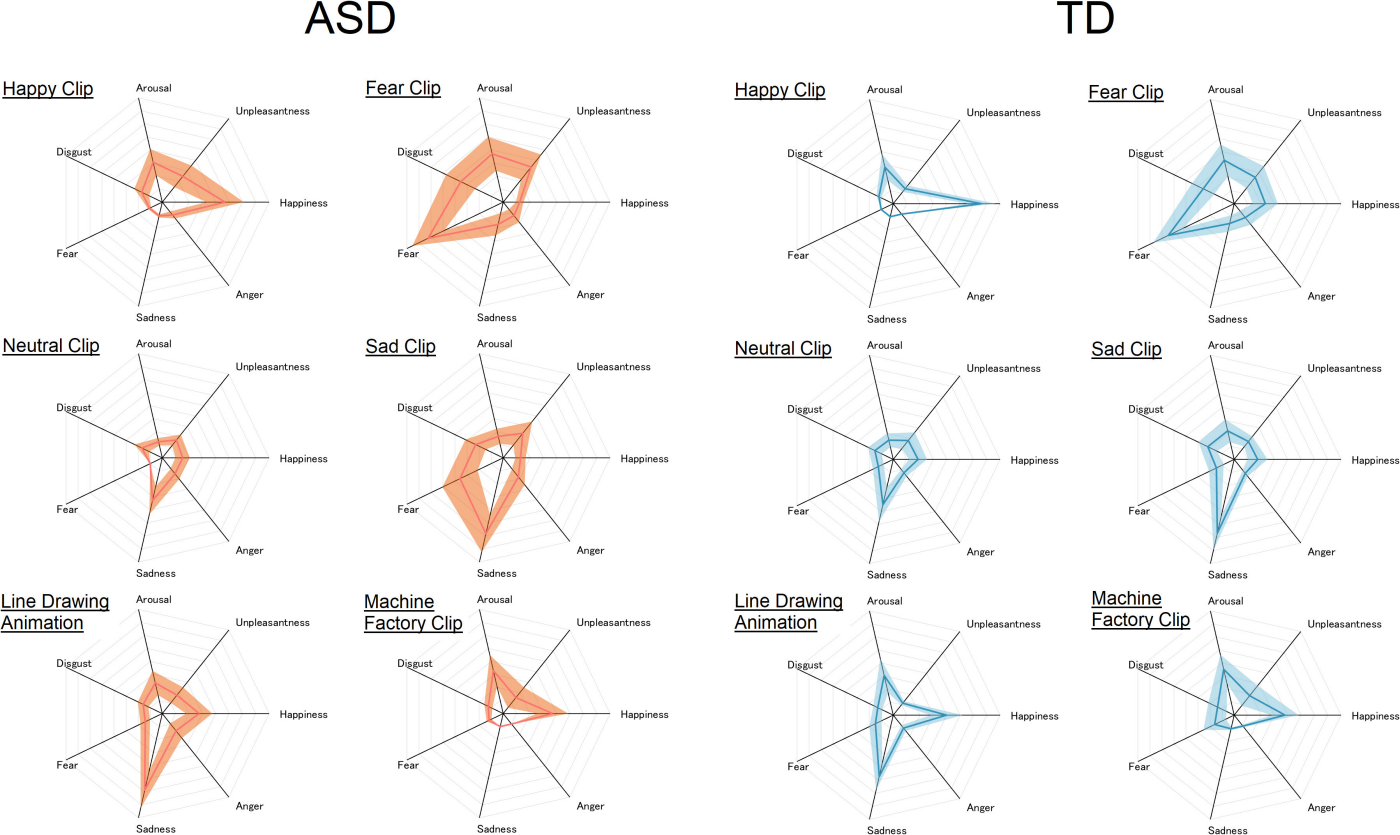
Subjective rating of each emotional state given to each of the six video clips in the ASD **(left)** and TD **(right)** groups. The center of the radar chart is zero, and the outermost point of each axis is eight. The thick line and the width of the shaded area along each axis represent mean and standard deviation, respectively.

The mean and standard deviation of the subjective ratings in each condition are shown in Figure 2.

The ANOVA revealed significant main effects of clip, *F*_(5, 180)_ = 14.53, *p* < 0.0001, $\eta_{\text{p}}^{2}$ = 0.29, and emotional state, *F*_(6, 216)_ = 30.53, *p* < 0.0001, $\eta_{\text{p}}^{2}$ = 0.46. There were also significant interactions between group and emotional state, *F*_(6, 216)_ = 4.08, *p* < 0.001, $\eta_{\text{p}}^{2}$ = 0.10, and between clip and emotional state, *F*_(30, 1, 080)_ = 35.04, *p* < 0.0001, $\eta_{\text{p}}^{2}$ = 0.49. All these main effects and interactions were further qualified by a significant three-way interaction between group, clip, and emotional state, *F*_(30, 1, 080)_ = 1.56, *p* = 0.028, $\eta_{\text{p}}^{2}$ = 0.042. No other effects reached significance, *F*s < 1.0, *p*s > 0.4. In order to clarify the source of the significant three-way interaction, two-way ANOVA with the between-participant factor of group (2) and within-participant factor of emotional state (7) was carried out for each clip.

#### Happy Clip

The ANOVA revealed a significant main effect of emotional state, *F*_(6, 216)_ = 59.67, *p* < 0.0001, $\eta_{\text{p}}^{2}$ = 0.62, and a significant interaction between group and emotional state, *F*_(6, 216)_ = 4.48, *p* = 0.0003, $\eta_{\text{p}}^{2}$ = 0.11. The main effect of group failed to reach significance, *F*_(1, 36)_ = 0.03, *p* = 0.85, $\eta_{\text{p}}^{2}$ < 0.001. Simple main effect analysis revealed significantly higher “happy” rating in TD than in the ASD group, *F*_(1, 36)_ = 5.72, *p* = 0.02, $\eta_{\text{p}}^{2}$ = 0.14. People with ASD also gave higher “unpleasantness” ratings than TD people, *F*_(1, 36)_ = 4.87, *p* = 0.034, $\eta_{\text{p}}^{2}$ = 0.12. A simple main effect of group failed to reach significance in the other emotional states, *F*s < 3.4, *p*s > 0.07.

#### Fear Clip

The main effect of emotional state reached significance, *F*_(6, 216)_ = 36.34, *p* < 0.0001, $\eta_{\text{p}}^{2}$ = 0.50. The main effect of group failed to reach significance, *F*_(1, 36)_ = 0.39, *p* = 0.53, $\eta_{\text{p}}^{2}$ = 0.01. The interaction between group and emotional state failed to reach significance either, *F*_(6, 216)_ = 1.88, *p* = 0.086, $\eta_{\text{p}}^{2}$ = 0.05.

#### Neutral Clip

The main effect of emotional state reached significance, *F*_(6, 216)_ = 13.43, *p* < 0.0001, $\eta_{\text{p}}^{2}$ = 0.27. Neither the main effect of group, *F*_(1, 36)_ = 0.20, *p* = 0.66, $\eta_{\text{p}}^{2}$ = 0.006, nor the interaction between group and emotional state, *F*_(6, 216)_ = 0.29, *p* = 0.94, $\eta_{\text{p}}^{2}$ = 0.01, reached significance.

#### Sad Clip

The main effect of emotional state, *F*_(6, 216)_ = 36.45, *p* < 0.0001, $\eta_{\text{p}}^{2}$ = 0.50, and the interaction between group and emotional state reached significance, *F*_(6, 216)_ = 3.36, *p* = 0.0035, $\eta_{\text{p}}^{2}$ = 0.085. The main effect of group failed to reach significance, *F*_(1, 36)_ = 0.93, *p* = 0.34, $\eta_{\text{p}}^{2}$ = 0.025. Simple main effect analysis revealed a significantly higher “fear” rating in the ASD than in the TD group, *F*_(1, 36)_ = 9.12, *p* = 0.005, $\eta_{\text{p}}^{2}$ = 0.20. A simple main effect of group reached significance for no other emotional state, *F*s < 1.9, *p*s > 0.17.

#### Line Drawing Animation

The main effect of emotional state reached significance, *F*_(6, 216)_ = 34.37, *p* < 0.0001, $\eta_{\text{p}}^{2}$ = 0.49. This main effect was qualified by a significant interaction between group and emotional state, *F*_(6, 216)_ = 2.62, *p* = 0.018, $\eta_{\text{p}}^{2}$ = 0.07. The main effect of group failed to reach significance, *F*_(1, 36)_ = 0.06, *p* = 0.80, $\eta_{\text{p}}^{2}$ = 0.002. Simple main effects of group failed to reach significance, *F*s < 3.5, *p*s > 0.07.

#### Machine Factory Clip

Only the main effect of emotional state was significant, *F*_(6, 216)_ = 22.34, *p* < 0.0001, $\eta_{\text{p}}^{2}$ = 0.38. Neither the main effect of group, *F*_(1, 36)_ = 0.61, *p* = 0.45, $\eta_{\text{p}}^{2}$ = 0.017, nor the interaction between group and emotional state reached significance, *F*_(6, 216)_ = 0.095, *p* = 0.99, $\eta_{\text{p}}^{2}$ = 0.003.

### Automatic Group Classification

We first tested whether the performance of the GB classifier was dependent on the number of PCs used as features. Thus, GB classifiers were trained and tested by using scores of 1–10 PCs as features. The PCs were entered into the classifier in descending order of the amount of variance explained. For example, when the number of PCs was two, the PCs with the first and second largest variance explained were used as the features. The performance of GB classifiers was compared by the accuracy and F1 score as performance indicator. The classifier showed its best performance when the number of PCs was eight.

The Mann–Whitney *U*-test revealed a significantly higher probability of being classified into ASD by the classifier in the ASD than in the TD group, *U* = 117, *p* = 0.037. When the threshold was set to 0.5, 13 out of 17 (76.4%) ASD participants and 8 out of 21 (38.1%) TD participants were classified into the ASD group. The proportions of participants classified into the ASD group differed significantly between the ASD and TD groups, $\chi_{(}^{2}1)$ = 5.59, *p* = 0.018.

Mean accuracy across 20 repetitions of cross-validation was 0.68 (SD = 0.015). Accuracy was significantly higher than chance (0.5), *t*_(_19) = 54.46, *p* < 0.001. Averages of sensitivity, specificity, precision, and F1 score were 0.76 (SD = 0.021), 0.63 (SD = 0.021), 0.63 (SD = 0.016), and 0.68 (SD = 0.015).

**Figure 3:**
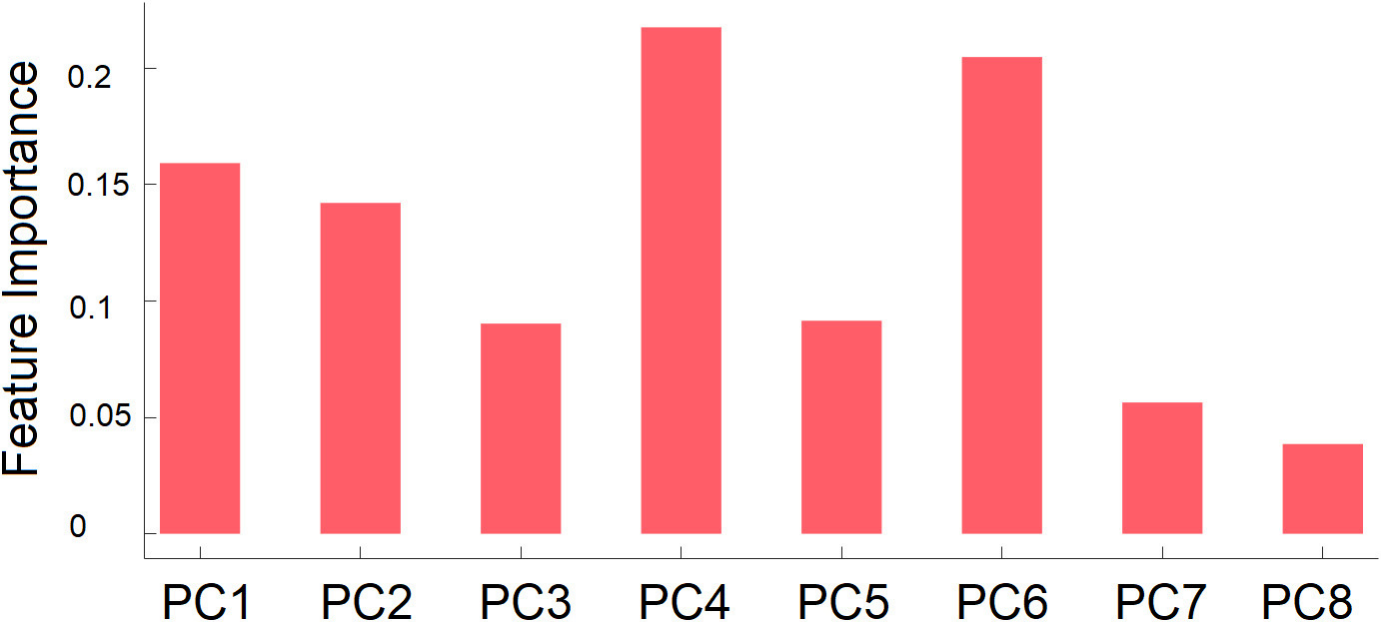
Mean feature importance of the right PCs used as features in group classification by GB classifier. PC*n* is the PC that explained the *n*-th largest amount of variance.

Feature importance of each PC is shown in Figure 3. As can be seen in this figure, the fourth and sixth PCs contributed most to the classification followed by the first and second PCs. For completeness, we compared scores of the eight PCs between the ASD and TD groups. No group difference was found, *t*s < 1.7, *p*s > 0.10.

### Relationship Between Classification Results and Scores of IRI and TAS-20

The average and standard deviations of IRI and TAS-20 are summarized in Table 3. Two ASD participants failed to complete TAS-20. In IRI, adults with ASD showed lower perspective-taking and higher personal distress scores than their neurotypical counterparts. The results also revealed a higher score in every factor of TAS-20 in the ASD than in the TD group.

**Table 3:** Mean and standard deviation of IRI and TAS-20 scores in each group, and the results of group comparison of each score by two-tailed *t*-test.

	**IRI**				**TAS-20**		
	**Perspective-taking**	**Empathic concern**	**Fantasy**	**Personal distress**	**Difficulty in identifying feelings**	**Difficulty in describing feelings**	**Externally oriented thinking**
ASD (*N* = 17)	18.0^*^	19.8	18.6	21.7^**^	19^**^	16.1^*^	21.7^**^
	(3.7)	(3.6)	(3.8)	(4.3)	(6.4)	(4.0)	(4.2)
TD (*N* = 21)	21.3	20.4	19.6	15.4	12.1	13.0	17.8
	(4.5)	(3.7)	(3.8)	(3.8)	(3.8)	(3.6)	(4.3)
*t*-value	−2.43	−0.5	−0.75	4.79	4.03	2.4	2.75
*df*	36	36	36	36	34	34	34
*p*	0.02	0.618	0.46	<0.001	<0.001	0.022	0.009

*In parenthesis are the standard deviations*.

* *p < 0.05*;

** *p < 0.01*.

The results of correlation coefficients between questionnaire results and the probabilities of being classified as ASD assigned by the GB classifier are summarized in Table 4. After significance threshold correction by Bonferroni's procedure, there was no significant correlation.

**Table 4:** Spearman's rank-order correlation coefficients between averaged probability and scores of IRI and TAS-20.

		**IRI**				**TAS-20**		
		**Perspective-taking**	**Empathic concern**	**Fantasy**	**Personal distress**	**Difficulty in identifying feelings**	**Difficulty in describing feelings**	**Externally oriented thinking**
All	Rho	−0.20	0.08	0.14	0.08	0.18	0.20	0.23
	*p*-value	0.236	0.634	0.414	0.643	0.287	0.235	0.182
ASD (*N* = 17)	Rho	−0.38	0.27	0.19	−0.11	−0.34	−0.25	0.54
	*p*-value	0.132	0.304	0.474	0.669	0.213	0.362	0.038
TD (*N* = 21)	Rho	0.13	−0.01	0.29	0.02	0.28	0.44	−0.10
	*p*-value	0.581	0.971	0.209	0.937	0.211	0.046	0.673

### Automatic Classification of Emotional States Within Each Group

The performance of the GB classifier was dependent on the number of PCs. Thus, we evaluated the performance of the GB classifier by changing the number of PCs in the same manner as described above. In the TD groups, the best performance was achieved when the number of PCs was six, while one in the ASD group. Thus, the number of PCs was set to these values.

The averaged accuracy was 0.34 in the TD group and 0.31 in the ASD group. The confusion matrix for each group is shown in Table 5 together with the hit rate, i.e., the probability that the classifier made a correct classification of each emotion.

**Table 5:** Confusion matrix of emotion categorization in each group.

**Group**	**Clip**	**Prediction**				**Hit rate (%)**
		**Happy**	**Fear**	**Neutral**	**Sad**	
ASD (*N* = 17)	Happy	**3**	8	2	4	17.6
	Fear	6	**6**	3	2	35.3
	Neutral	4	4	**7**	2	23.5
	Sad	5	2	5	**5**	29.4
TD (*N* = 21)	Happy	**10**	4	2	5	47.6
	Fear	5.1	**6.9**	4.4	4.6	24.3
	Neutral	4.8	2.6	**6.85**	6.75	22.9
	Sad	4.8	2.85	8.25	**5.1**	22.9

*The number in each cell represents averaged frequency. Hit rates are also shown in the rightmost column. The numbers along the diagonal line (bold) represent averaged frequency of hit*.

The cosine similarities between each pair of emotional states are listed in Table 6. As can be seen in this table, cosine similarities between happy and neutral, and between happy and sad, were relatively low compared with the other pairwise combinations in both ASD and TD groups.

**Table 6:** Mean cosine similarity between emotional states in each group.

		**Happy**	**Fearful**	**Neutral**	**Sad**
ASD	Happy	1	0.9	0.74	0.71
	Fearful		1	0.86	0.82
	Neutral			1	0.89
	Sad				1
TD	Happy	1	0.85	0.79	0.75
	Fearful		1	0.87	0.85
	Neutral			1	0.97
	Sad				1

*The number in each cell represents averaged cosine similarity*.

## Discussion

Recent advances in image processing technology have enabled the quantification of ANS activation by non-contact measurement of pulse waves from images of the skin surface (30, 31, 44). Here, we used this technology to measure ANS activity in adults with ASD in response to six types of video clips.

At the behavioral level, ASD people gave lower “happy” rating (for happy clip) and higher “fear” (for sad clip) and “unpleasant” (for happy clip) ratings than TD people. The overall pattern indicates a stronger negativity bias in emotional reactions to video clips in ASD individuals. Anxiety disorder is a common comorbidity of ASD (24, 48) and sometimes leads to misdiagnosis of patients with mood disorders as ASD (7). The present finding of negativity bias in individuals with ASD is in line with this clinical observation. At the same time, the present results tell nothing about the cause of this bias. This could be due to hyperactivation in neural centers of emotion induction, such as the amygdala and anterior cingulate cortex (49), or the atypical interpretation of the physiological state induced by video clips.

We tried a semiautomatic classification of TD and ASD groups by submitting the features reflective of ANS activation into a gradient boosting classifier. The classifier succeeded in classifying unknown participants into either the TD or ASD group above the chance level. Furthermore, the probability of belonging to the ASD group estimated by the best performance classifier was significantly higher in the ASD group than in the TD group, which further supports the proposition that non-contact measurement of pulse waves captures atypical responses useful for classifying ASD from TD adults.

At the same time, we have to point out that our success was only modest. Although the accuracy rate was significantly above chance, the values of performance indicators were far from being acceptable for practical use. The relatively poor performance of the classifier is not surprising considering the fact that we focused on a single facet of peripheral nervous system activation, i.e., pulse wave. Needless to say, faces covey abundant information, and some of the previous studies on digital phenotyping of ASD made efficient use of facial information in automatic discrimination of ASD and TD samples (10, 11, 50). Thus, a combination of the other sources of facial information together with non-contact measurement of pulse waves might lead to the establishment of a classifier with better prediction performance.

The relatively low performance of our classifier is also attributable to heterogeneity in ASD participants. It is now well-acknowledged that people with ASD are a heterogeneous group and the symptomatic profiles differ greatly among patients (51). This observation gained further support from the emerging view that each symptom of ASD derives from a different set of etiological factors (52, 53). Given this, it is highly possible that participants in our ASD group had heterogeneous characteristics, which might have yielded a variance in ANS responses to emotional clips. Of particular relevance to the present study, it is possible that participants with hypersensitivity to visual and auditory stimulation might have shown different responses to the video clips from those without hypersensitivity in the present study. Unfortunately, the sample size of the present study is not large enough to examine the effects of symptomatic heterogeneity on physiological responses to emotional clips. This point is surely an important topic for future study.

The results of self-administered questionnaires revealed lower perspective-taking in ASD. Considering that the vicarious experience of the characters' emotion is at the core of emotional experience in film viewing, one might conceive that a lower tendency of perspective-taking might explain a different pattern of ANS activation in ASD compared with TD participants. However, the results of correlation analysis do not support this view; correlation analyses did not show a reliable association between IRI scores and averaged probability belonging to the ASD group generated by the classifier. All of the three scores measured by TAS-20 were higher in the ASD group, which is consistent with the reported strong alexithymic tendency in ASD (54).

The classification results of basic emotional states revealed comparable accuracy in the classification of four emotional states by gradient boosting classifier in the TD and ASD groups. The averaged accuracy rate of classification across all the emotional categories was numerically low in the ASD than in the TD group, but this is probably because the sample size of adults with ASD was smaller than that of TD adults; the classifier for ASD was disadvantaged due to the smaller size of the training dataset. In both ASD and TD groups, cosine similarity was high for the combination between happy and fear, but was relatively low for the combinations of happy and two low arousal emotions (neutral, sad). This indicates that indices extracted from pulse wave are sensitive to arousal level, consistent with previous studies that show the association between arousal state and ANS activation (20). Interestingly, hit rate for happy emotion was drastically lower in the ASD than in the TD group. Actually, the pattern of ANS activation induced by the happy clip was more likely to be confused with that induced by the fearful clip in the ASD than in the TD group. This might indicate a less clear differentiation of emotional state in ASD (47). This finding also dovetails with the proposal that people with ASD show atypical pattern of activation in fear-related neural circuits (18, 55) by emotional stimulation. However, definitive conclusion awaits further empirical studies. An alternative explanation for this result is that the film set used in the present study was not optimized to induce discrete emotional states in ASD participants. Given the prevalent use of films for emotion induction (34, 56) in TD population, it is desirable to establish a standardized set of emotion-inducing films suitable for people with ASD.

The present study showed the potential of non-contact ANS measurement in the automatic classification of adult males with and without ASD. The number of studies reporting the successful application of affordable non-contact measurement to digital phenotyping of atypical behavior in ASD has increased recently (8, 11–13). Combined with these previously established methodologies, the current technique could serve as an assessment tool to screen hitherto undiagnosed or misdiagnosed adults with ASD (5–7). However, there are several limitations that qualify our conclusion. First, many of the relevant studies, including the current one, fail to include participants with other psychiatric conditions, and as such, it remains uncertain whether non-contact measurement of pulse wave can discriminate ASD from patients with other psychiatric conditions. Second, the present study recruited only adult males with and without ASD. Thus, it is unclear whether the current technology could contribute to the classification of females or pediatric samples with ASD from their neurotypical counterparts. Third, the distribution of ASD and TD people in the present study does not mirror the prevalence of ASD in the population. The prevalence of ASD among males is estimated to be around 1 in 50–60 (57), though the reported number varies widely among studies. Given the relatively low prevalence rate, the sensitivity/specificity of our classifier is unclear when it is applied to actual clinical settings. Fourth, we used a video camera with a 30-Hz frame rate for cost-effectiveness. The use of a low frame rate might have made the estimation of some of the ANS activation indicators somewhat unreliable. In other words, the performance of the classifier might be improved by integrating video cameras with a higher frame rate. Considering these, it would be desirable to test the reliability of the current technique by recruiting a more diverse population and using more suitable equipment in future studies.

## Data Availability Statement

The raw data supporting the conclusions of this article will be made available by the authors, without undue reservation.

## Ethics Statement

The studies involving human participants were reviewed and approved by The ethical committee of Graduate School of Biomeidcal Sciences in Nagasaki University. The patients/participants provided their written informed consent to participate in this study.

## Author Contributions

HD, NT, and CK conceived of the research. HD, CK, KM, RM, and TN collected data. NT, KM, RM, and TN carried out the analysis of video images. HD analyzed the data and wrote the manuscript. All authors contributed to the article and approved the submitted version.

## Conflict of Interest

The authors declare that the research was conducted in the absence of any commercial or financial relationships that could be construed as a potential conflict of interest.

## Footnotes

^1^The 13 films tested included “*Lullaby of Narayama* (1983)” (sad), “*Dark Water* (Japanese Version, 2002)” (fear), “*A Tale of Two Sisters* (2003)” (fear), “*Gottu-Ee-Kanji* (Japanese TV comedy show)” (an episode other than the one used in the experiment; happy), and “*Maboroshi* (1995)” (neutral) and a video of machine factory (other than the one used in the experiment) in addition to six clips selected.
